# Misuse of *reporter score* in microbial enrichment analysis

**DOI:** 10.1002/imt2.95

**Published:** 2023-02-23

**Authors:** Lei Liu, Ruixin Zhu, Dingfeng Wu

**Affiliations:** ^1^ The Shanghai Tenth People's Hospital, School of Life Sciences and Technology Tongji University Shanghai China; ^2^ National Clinical Research Center for Child Health, The Children's Hospital Zhejiang University School of Medicine Hangzhou Zhejiang China

## Abstract

A modified new method for microbial enrichment analysis, *reporter score* was incorrectly used in many articles due to a lack of comprehensive and systematic understanding of the original method by the researchers, leading to a serious snowball effect. Here we describe the reasons for the misuse of reporter score and its negative impact on microbial research and hope this comment will facilitate community discussion on the importance of statistical rigor, informing future efforts to enhance reliable and reproducible research.

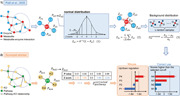

Enrichment analysis has become a common technique in bioinformatics studies for its advantages in reducing complexity and enhancing explanatory power [1]. Many methods have been proposed to facilitate the biological interpretation of expression data, including the hypergeometric test, Fisher's exact test, and Gene Set Enrichment Analysis [2], especially for transcriptome research [3]. Along with the explosive growth of metagenome data, various enrichment approaches have been implemented in microbial research to gain comprehensive and in‐depth knowledge of microbial functions. It has been noticed that besides the traditional methods aforementioned, a novel hypothesis‐driven method called *reporter score*, which was originally developed to reveal the transcriptional regulatory patterns in metabolic networks [4, 5], has been introduced into microbial research for pathway enrichment analysis. However, we found that many microbiome studies [6, 7, 8] misused *reporter scores*, including using the method for the wrong purpose and misinterpreting the results, leading to misleading conclusions and serious snowball effects. In light of these observations, there is a desperate need to re‐examine the utilization of *reporter score* in microbial pathway enrichment analysis and analyze the causes of misuse, pointing to the importance of statistical rigor in bioinformatics studies, especially when modifying and migrating developed methods to new research areas. Our aim is to raise awareness within the research community to avoid the misuse of the *reporter score* algorithm as early as possible, thereby mitigating the snowball effect, and putting forward suggestions to circumvent the recurrence of similar problems.

## MISUSE OF *REPORTER SCORE* LEADS TO MISLEADING CONCLUSIONS IN MICROBIAL RESEARCH

**Figure 1:**
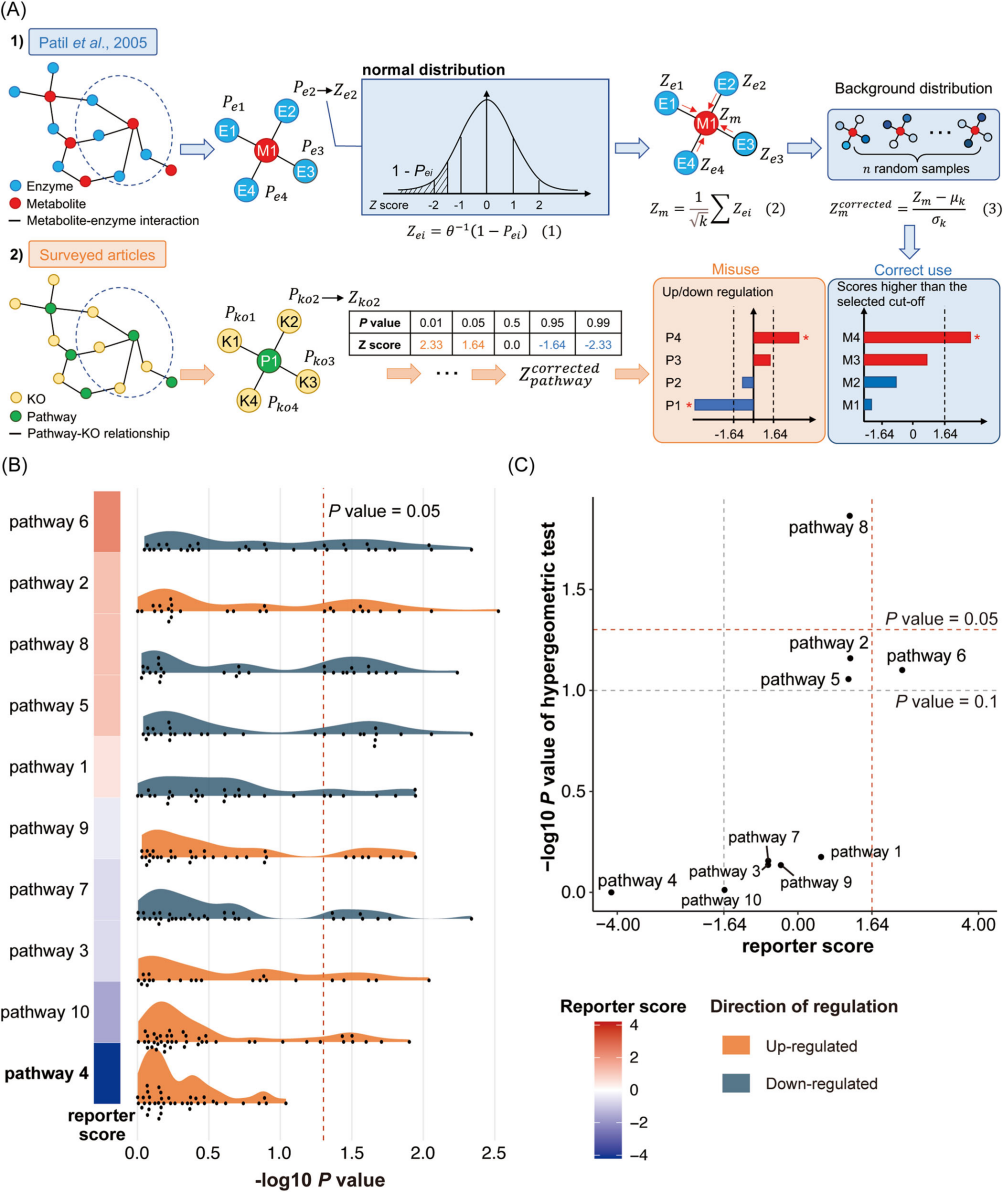
Misuse of *reporter score* leads to misleading conclusions. (A) Illustration of the *reporter score* algorithm utilized to: (1) identify reporter metabolites in the original method paper and (2) determine significantly enriched pathways in surveyed articles. In bar charts, red and blue bars indicate positive and negative reporter scores, respectively. (B) Distribution of *P* values of KOs for each pathway and their corresponding reporter scores. KOs are represented by dots. Dashed line indicates a *P* value of 0.05. (C) Plot shows the reporter score versus −log_10_ *P* value from the hypergeometric test. Vertical dashed lines indicate a reporter score of ±1.64. Horizontal dashed lines indicate *P* values of 0.05 and 0.1. KEGG, Kyoto Encyclopedia of Genes and Genomes; KOs, KEGG orthologies.

The *reporter score* algorithm was first developed by Patil and Nielsen [5] in 2005 for the identification of so‐called reporter metabolites that represents hot spots in terms of metabolic regulation (Figure 1A). By leveraging differential transcription data, Student's *t* test is performed on all the enzyme nodes of the metabolic graph to obtain *P* values. For each enzyme node, the $P_{\mathrm{ei}}$ is then converted to a Z score ($Z_{\mathrm{ei}}$) by using the inverse normal cumulative distribution $\theta^{-1}$. To identify reporter metabolites, each metabolite node is scored based on the size‐independent aggregated Z scores of its k neighboring enzymes. The background distribution is corrected by subtracting the mean $\mu_{k}$ and dividing by the standard deviation $\sigma_{k}$ of the aggregated Z scores of several sets of k enzymes chosen randomly from the metabolic graph. The authors reasoned that the metabolites with $Z_{m}^{\mathrm{corrected}}$ above the selected cut‐off (e.g., 1.64 in Figure 1A, corresponding to a *P* value of 0.05 converted by the normal cumulative distribution function) are defined as reporter metabolites.

In recent years, researchers have migrated the *reporter score* algorithm to microbial enrichment analysis to detect significantly enriched pathways, replacing enzyme nodes with Kyoto Encyclopedia of Genes and Genomes (KEGG) orthologies (KOs) and metabolic subnetworks with KEGG pathways or modules (Equations (1), (2), (3), Figure 1A).

(1) $$Z_{{\mathrm{KO}}_{i}}=\theta^{-1}\left(1-P_{{\mathrm{KO}}_{i}}\right),$$

(2) $$Z_{\mathrm{pathway}}=\frac{1}{\sqrt{k}}\sum Z_{{\mathrm{KO}}_{i}},$$

(3) $$Z_{\mathrm{pathway}}^{\mathrm{corrected}}=\frac{Z_{\mathrm{pathway}}-\mu_{k}}{\sigma_{k}}.$$

Here the *P* values of each KO can be computed using the significance testing method, and k is the number of KOs involved in the pathway (or module). In contrast to the original method paper [5], in several studies [6, 7, 8], pathways with the *absolute* value of reporter score greater than the selected level of significance (e.g., $\left|Z_{\mathrm{pathway}}^{\mathrm{corrected}}\right|\;>\;1.64$) were regarded as significantly enriched pathways, and researchers determined the regulation direction of pathways (e.g., up/downregulation with respect to reference condition) according to the sign of reporter score (Figure 1A). For example, Feng et al. [6] demonstrated that the KEGG module for transporting the amino acid histidine was enriched in carcinoma compared with adenoma, with a reporter score >1.7. Conversely, the KEGG module for synthesizing histidine was enriched in adenoma samples, taking into account that the reporter score was less than −1.7. From a statistical perspective, however, such interpretations are mistaken. *The reporter score is undirected*—the plus or minus sign of the reporter score does not reflect the regulation direction. The Z score converted from the *P* value does not contain the up/downregulation information of the KOs, but the probability information supporting the null hypothesis depends on the significance estimation method used. In particular, according to the inverse normal cumulative distribution (Equation 1), the negative Z score is converted from a *P* > 0.5, which indicates that the corresponding KO is not differentially enriched, rather than that the KO is downregulated in the treatment group (Figure 1A). Thus, the *reporter score* algorithm described here is an enrichment method disregardful of the up/downregulation information of KOs in the pathway, and it is rather incorrect to consider the sign of the reporter score as the regulation direction of the pathway [6, 7, 8].

To illustrate the idea, we created a set of 200 KOs and 10 pathways as simulated data (Table S1). The *P* value of each KO was randomly generated from a uniform distribution, and 50 KOs were significantly differentially expressed. Each pathway contains a random number (k) of KOs. To better explain that the reporter score is nondirectional, we artificially set that KOs in the same pathway have consistent directionality of regulation. For each pathway, the aggregated Z score of the k KOs involved was computed and then corrected for the background distribution. Evidently, the pathway with the lowest reporter score was predominantly made up of the least significant KOs (pathway 4, Figure 1B), suggesting its negligible response to a perturbation, which, notably, is independent of the expression directionality (i.e., upregulation of KOs in pathway 4, Figure 1B). However, by leveraging a common visualization of reporter score in surveyed articles and cut‐offs of ±1.64, pathway 4 was misinterpreted as a significantly downregulated pathway (Figure 1A). Needless to say, misinterpretation of the reporter score will cause misleading results in pathway enrichment analysis, for example, taking unimportant pathways as significantly downregulated pathways and considering all pathways with positive reporter scores upregulated, which may impede and misdirect microbial research, such as studies of microbiome in disease pathogenesis.

Compared with the hypergeometric test, which only considers the predefined list (such as KOs/genes with *P* < 0.05), the *reporter score* considers all KOs involved in the pathway so that it can synthetically measure the gene differences in the pathway and is not affected by a priori cut‐off of gene significance [5]. Notably, although these two methods are divergent, the results of the *reporter score* algorithm were similar to those of the hypergeometric test (Figure 1C). For example, pathways with higher reporter scores were also marked as enriched by the hypergeometric test, and pathway 4 was not significantly enriched because the *P* values of its relevant KOs were greater than 0.05 (Figure 1B). Similar results were also found in the demo data set (Figure S1A), a nonalcohol fatty liver disease cohort containing 72 patients with mild/moderate fibrosis and 14 patients with advanced fibrosis [9].

## MISUSING *REPORTER SCORE* HAS CAUSED SNOWBALL EFFECT IN MICROBIOME FIELD

**Figure 2:**
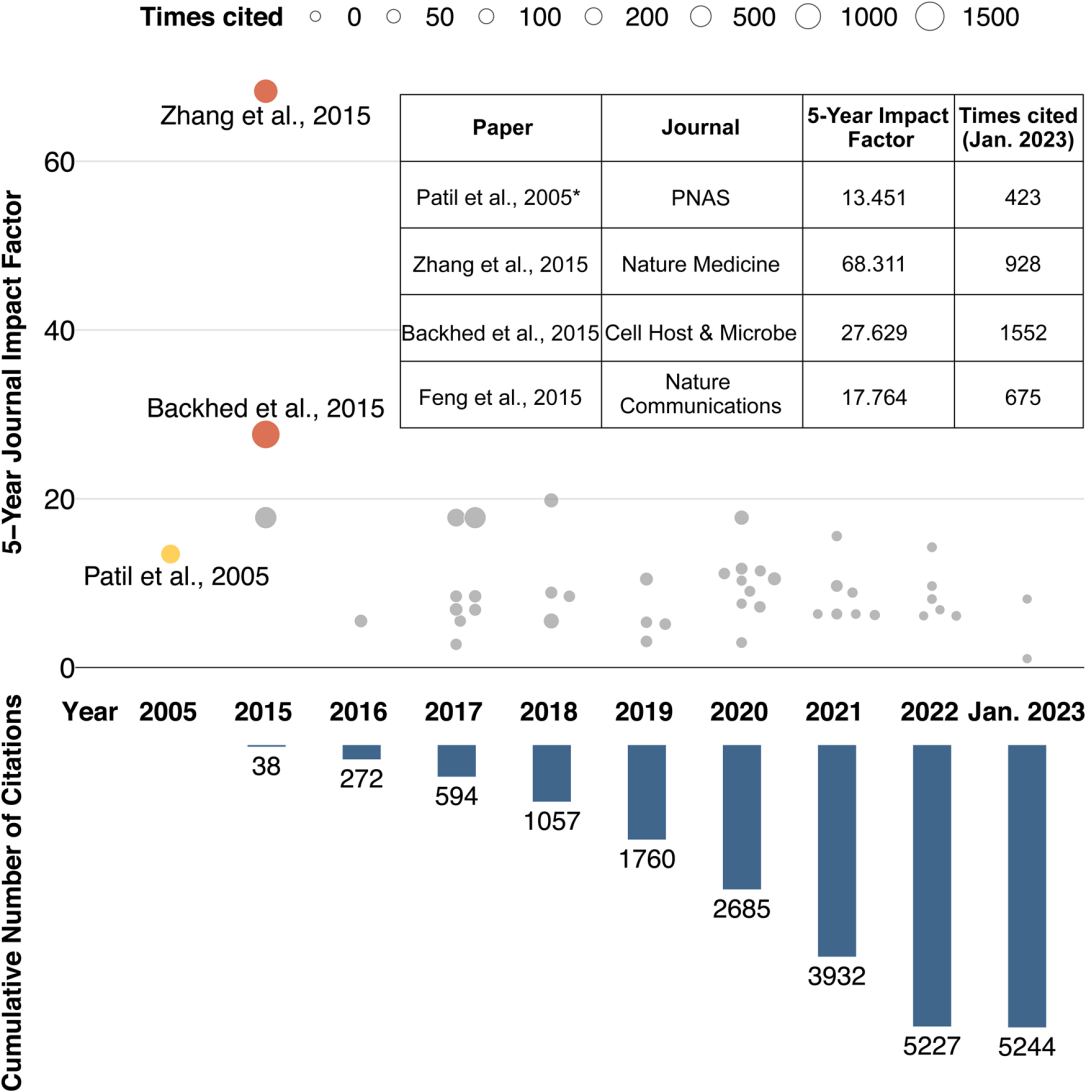
Misuse of *reporter score* has caused snowball effect in microbiome field. Information on publications citing the original method paper and misusing the *reporter score* algorithm, and the cumulative number of citations from 2015 to 2023. Each dot represents a paper. The original method paper is highlighted in yellow, and surveyed articles published in journals with a 5‐year impact factor higher than 20 are highlighted using red color. The embedded table presents the details of the original paper and the three articles published in 2015. *The original method paper. PNAS, Proceedings of the National Academy of Sciences.

Since *reporter score* was first misused for microbial enrichment analysis in 2015 [6], similar misuse has thus far been found in at least 45 papers, causing a deleterious impact (Figure 2). Surveyed papers were manually collected from citations of the original method paper. According to Figure 2, although the number of papers misusing *reporter scores* did not increase significantly over time, the influence of these papers (i.e., the cumulative number of citations) showed an exponential growth trend. Among surveyed papers, Zhang et al. [8] and Bäckhed et al. [7] have a greater impact on relevant studies due to their published year and high journal impact factor. It is important to point out that although most surveyed articles cited the original method paper in 2005 [5], from the method description, it can be deduced that the enrichment analysis of these works was inspired by the three articles published in 2015 [6, 7, 8]. Such irregular citation, in combination with a half‐baked knowledge of *reporter score*, was a major factor contributing to the subsequent misuses.

Most strikingly, as far back as 2008, Patil and Nielsen proposed a workflow called *Reporter Feature* analysis whereby the regulation directionality and statistical significance can be determined simultaneously [3, 4]. The ranking of desired features is compared by synthesizing the results from the whole list of KOs and the information on up/downregulation, providing in‐depth insights into the pathogenesis of the disease studied or the biological role of a perturbation of interest. This workflow has been applied to a broad range of scientific topics, including microbial research. For instance, by leveraging *Reporter Feature* analysis, Karlsson et al. [10] identified reporter pathways in the metagenomes of women with type 2 diabetes. In our demo data set, *Reporter Feature* achieved results of up‐ and downregulated enrichment, which was an important complement to the *reporter score* (Figure S1). Hence, we hold that had surveyed papers taken a deep dive into the latest development in *reporter score* algorithm, misuses, and snowball effects could have been avoided. In addition, other commonly used methods for microbial enrichment analysis were listed in Table S3 for the convenience of researchers.

## CONCLUSIONS

In recent years, the rapid development of bioinformatics has played a tremendous role in promoting research in scientific fields, such as transcriptomics, proteomics, microbiomics, and so forth [11, 12]. Behind this boom, however, lies the seeds of risks. First, as an interdisciplinary field, bioinformatics requires various professional knowledge and has high barriers to entry, which hinders the development of novel analytical methods. Also, the lack of outstanding bioinformatics talents and the neglect of basic statistics and mathematics leads to the decline of analysis quality and nonnegligible damage to research, especially for scientific fields in the stage of rapid development, such as microbiome.

Misinterpretation or misuse of statistical techniques will cause problems of unreliability and reproducibility, which have been underlined over a wide range of scientific disciplines [13, 14]. It has been noticed that errors often occur if researchers migrate a developed statistical approach into a new field without fully understanding the assumption and logic beyond the approach. Such ill‐informed practice may eventuate insidious issues, for example: (1) applying the method to problems that it is not applicable to; (2) misinterpretation of results; and (3) unfounded modification and extension of the method. Moreover, a series of errors resulting from citing problematic articles highlights a common problem in scientific research—when referencing a method, primary researchers may be addicted to the authority of papers (i.e., impact factor and the number of citations) but ignoring the accuracy of the method. The misuse of the reporter algorithm is a representative example of these problems. All the surveyed articles misinterpreted pathway reporter scores, especially the negative ones, although their calculation processes were correct. Interpreting *reporter scores* under habitual thinking and adopting a published method on faith without comprehending its statistical meaning are the underlying causes of why such a naïve error kept being made. Due to the importance of microbial enrichment analysis in metagenomics research, the misuse of *reporter scores* will lead to partial misunderstanding about the enrichment of biological functional pathways. Meanwhile, the papers misusing *reporter scores*, especially those of pretty impact, are likely to cause the same mistake in the subsequent research that quotes them. As the development of microbiomes skyrockets, the number of publications has increased staggeringly, which may further aggravate the snowball effect of misuse, inducing a highly adverse impact on microbial research.

The existing review system is stringent for the reliability and reproducibility of experimental research, and many journals have developed a checklist to ensure that published papers provide sufficient information for readers to reproduce the work [15]. Nevertheless, the documentation of data processing, analysis, and statistical algorithms in publications is usually not sufficiently detailed for bioinformatics researches. To augment the reproducibility and reliability of research, therefore, we suggest that the composition of reviewers should be more reasonable, such as selecting reviewers from different fields, and the review system of journals needs to create a specialized checklist for bioinformatics research. The AIMe registry, a community‐driven registry for artificial intelligence in biomedical research, is a potential referential scheme [16]. Besides, multidisciplinary collaboration, particularly with statisticians, should be encouraged to promote rigorous research. Last but not least, strengthening the training of bioinformaticians with solid basic statistical and mathematical knowledge will guarantee the significant and stable development of bioinformatics, facilitating scientific research in various domains.

## AUTHOR CONTRIBUTIONS

Lei Liu did the literature search, analyzed the results, and wrote the manuscript. Ruixin Zhu provided suggestions for the analysis and contributed to the manuscript. Dingfeng Wu conceptualized the study and wrote the manuscript. All authors read and approved the final manuscript.

## CONFLICT OF INTEREST STATEMENT

The authors declared no conflict of interest.

## Supporting information

Supplementary information.

Supplementary information.

## Data Availability

All data generated or analyzed during this study are included in the supplementary information files. Statistical analysis and data visualization were performed using the R software version 4.1.1. All the software packages used in this study are open source and publicly available, and the code and data used in this study are available on GitHub at https://github.com/dfwlab/ReporterScore. Supplementary materials (figures, tables, scripts, graphical abstract, slides, videos, Chinese translated version, and update materials) may be found in the online DOI or iMeta Science http://www.imeta.science/.

## References

[1] Khatri, Purvesh , Marina Sirota , and Atul J. Butte . 2012. “Ten Years of Pathway Analysis: Current Approaches and Outstanding Challenges.” PLoS Computational Biology 8: e1002375. 10.1371/journal.pcbi.1002375 22383865 PMC3285573

[2] Subramanian, Aravind , Pablo Tamayo , Vamsi K. Mootha , Sayan Mukherjee , Benjamin L. Ebert , Michael A. Gillette , Amanda Paulovich , et al. 2005. “Gene Set Enrichment Analysis: A Knowledge‐Based Approach for Interpreting Genome‐Wide Expression Profiles.” Proceedings of the National Academy of Sciences 102: 15545–50. 10.1073/pnas.0506580102 PMC1239896 16199517

[3] Väremo, Leif , Jens Nielsen , and Intawat Nookaew . 2013. “Enriching the Gene Set Analysis of Genome‐Wide Data by Incorporating Directionality of Gene Expression and Combining Statistical Hypotheses and Methods.” Nucleic Acids Research 41: 4378–91. 10.1093/nar/gkt111 23444143 PMC3632109

[4] Oliveira, Ana Paula , Kiran Raosaheb Patil , and Jens Nielsen . 2008. “Architecture of Transcriptional Regulatory Circuits Is Knitted Over the Topology of Bio‐molecular Interaction Networks.” BMC Systems Biology 2: 17. 10.1186/1752-0509-2-17 18261202 PMC2268660

[5] Patil, Kiran Raosaheb , and Jens Nielsen . 2005. “Uncovering Transcriptional Regulation of Metabolism by Using Metabolic Network Topology.” Proceedings of the National Academy of Sciences 102: 2685–89. 10.1073/pnas.0406811102 PMC549453 15710883

[6] Feng, Qiang , Suisha Liang , Huijue Jia , Andreas Stadlmayr , Longqing Tang , Zhou Lan , and Dongya Zhang , et al. 2015. “Gut Microbiome Development Along the Colorectal Adenoma–Carcinoma Sequence.” Nature Communications 6: 6528. 10.1038/ncomms7528 25758642

[7] Bäckhed, Fredrik , Josefine Roswall , Yangqing Peng , Qiang Feng , Huijue Jia , Petia Kovatcheva‐Datchary , Yin Li , et al. 2015. “Dynamics and Stabilization of the Human Gut Microbiome During the First Year of Life.” Cell Host & Microbe 17: 690–703. 10.1016/j.chom.2015.04.004 25974306

[8] Zhang, Xuan , Dongya Zhang , Huijue Jia , Qiang Feng , Donghui Wang , Di Liang , Xiangni Wu , et al. 2015. “The Oral and Gut Microbiomes Are Perturbed in Rheumatoid Arthritis and Partly Normalized After Treatment.” Nature Medicine 21: 895–905. 10.1038/nm.3914 26214836

[9] Loomba, Rohit , Victor Seguritan , Weizhong Li , Tao Long , Niels Klitgord , Archana Bhatt , Parambir Singh Dulai , et al. 2017. “Gut Microbiome‐Based Metagenomic Signature for Non‐invasive Detection of Advanced Fibrosis in Human Nonalcoholic Fatty Liver Disease.” Cell Metabolism 25: 1054–62. 10.1016/j.cmet.2017.04.001 28467925 PMC5502730

[10] Karlsson, Fredrik H. , Valentina Tremaroli , Intawat Nookaew , Göran Bergström , Carl Johan Behre , Björn Fagerberg , Jens Nielsen , and Fredrik Bäckhed . 2013. “Gut Metagenome in European Women with Normal, Impaired and Diabetic Glucose Control.” Nature 498: 99–103. 10.1038/nature12198 23719380

[11] Rao, Anjali , Dalia Barkley , Gustavo S. França , and Itai Yanai . 2021. “Exploring Tissue Architecture Using Spatial Transcriptomics.” Nature 596: 211–20. 10.1038/s41586-021-03634-9 34381231 PMC8475179

[12] Hanahan, Douglas . 2022. “Hallmarks of Cancer: New Dimensions.” Cancer Discovery 12: 31–46. 10.1158/2159-8290.Cd-21-1059 35022204

[13] Begley, C. Glenn , and John P. A. Ioannidis . 2015. “Reproducibility in Science.” Circulation Research 116: 116–26. 10.1161/CIRCRESAHA.114.303819 25552691

[14] Baker, Monya . 2016. “1,500 Scientists Lift the Lid on Reproducibility.” Nature 533: 452–4. 10.1038/533452a 27225100

[15] Voelkl, Bernhard , Naomi S. Altman , Anders Forsman , Wolfgang Forstmeier , Jessica Gurevitch , Ivana Jaric , Natasha A. Karp , et al. 2020. “Reproducibility of Animal Research in Light of Biological Variation.” Nature Reviews Neuroscience 21: 384–93. 10.1038/s41583-020-0313-3 32488205

[16] Matschinske, Julian , Nicolas Alcaraz , Arriel Benis , Martin Golebiewski , Dominik G. Grimm , Lukas Heumos , Tim Kacprowski , et al. 2021. “The AIMe Registry for Artificial Intelligence in Biomedical Research.” Nature Methods 18: 1128–31. 10.1038/s41592-021-01241-0 34433960

