# Supplementary material for: Misuse of reporter score in microbial enrichment analysis
Source: Imeta. 2023 Feb 23;2(2):e95. doi: 10.1002/imt2.95 (PMC10989806; doi:10.1002/imt2.95)
Supplement: Supplementary file 1 — Supplementary information. [file IMT2-2-e95-s002.docx]

**Supplementary Figures**

**Figure S1. *Reporter score* and *Reporter Feature* analysis on the demo dataset.** A) Plot shows the reporter score versus -log_10_ *P* value from hypergeometric test. Vertical dashed lines indicate reporter score of ±1.64. Horizontal dashed lines indicate *P* values of 0.05 and 0.1. B) The heatmap presents the top five pathways for each directionality class in *Reporter Feature* analysis. The *P* value of each pathway is printed inside each cell. The *P* value represents the significance of a reporter score, and it is estimated using the normal cumulative distribution function, $P=1-\theta(Z_{corrected})$. The non-directional class (i.e., *reporter score*) reflects the differential expression in general and does not take expression directionality into account. The mixed-directional class provides information of the separate subsets of up- and down-regulated genes. Pathways containing important components of up regulation can have important components of down regulation simultaneously. The distinct-directional class uncovers whether the gene set is coordinately up-regulated or down-regulated. C) The plot compares the corrected non-directional *reporter score* and -log_10_ *P* values of mixed-directional up and down classes. The same pathway is connected by a line.
